# Dehydrogenation, disproportionation and transfer hydrogenation reactions of formic acid catalyzed by molybdenum hydride compounds†
†Electronic supplementary information (ESI) available. CCDC 1028432. For ESI and crystallographic data in CIF or other electronic format see DOI: 10.1039/c4sc03128h



**DOI:** 10.1039/c4sc03128h

**Published:** 2015-01-14

**Authors:** Michelle C. Neary, Gerard Parkin

**Affiliations:** a Department of Chemistry , Columbia University , New York , New York 10027 , USA . Email: parkin@columbia.edu

## Abstract

The cyclopentadienyl molybdenum hydride compounds, Cp^R^Mo(PMe_3_)_3–*x*_(CO)_*x*_H (Cp^R^ = Cp, Cp*; *x* = 0, 1, 2 or 3), are catalysts for the dehydrogenation of formic acid, with the most active catalysts having the composition Cp^R^Mo(PMe_3_)_2_(CO)H. The mechanism of the catalytic cycle is proposed to involve (i) protonation of the molybdenum hydride complex, (ii) elimination of H_2_ and coordination of formate, and (iii) decarboxylation of the formate ligand to regenerate the hydride species. NMR spectroscopy indicates that the nature of the resting state depends on the composition of the catalyst. For example, (i) the resting states for the CpMo(CO)_3_H and CpMo(PMe_3_)(CO)_2_H systems are the hydride complexes themselves, (ii) the resting state for the CpMo(PMe_3_)_3_H system is the protonated species [CpMo(PMe_3_)_3_H_2_]^+^, and (iii) the resting state for the CpMo(PMe_3_)_2_(CO)H system is the formate complex, CpMo(PMe_3_)_2_(CO)(κ^1^-O_2_CH), in the presence of a high concentration of formic acid, but CpMo(PMe_3_)_2_(CO)H when the concentration of acid is low. While CO_2_ and H_2_ are the principal products of the catalytic reaction induced by Cp^R^Mo(PMe_3_)_3–*x*_(CO)_*x*_H, methanol and methyl formate are also observed. The generation of methanol is a consequence of disproportionation of formic acid, while methyl formate is a product of subsequent esterification. The disproportionation of formic acid is a manifestation of a transfer hydrogenation reaction, which may also be applied to the reduction of aldehydes and ketones. Thus, CpMo(CO)_3_H also catalyzes the reduction of a variety of ketones and aldehydes to alcohols by formic acid, *via* a mechanism that involves ionic hydrogenation.

## Abstract 2

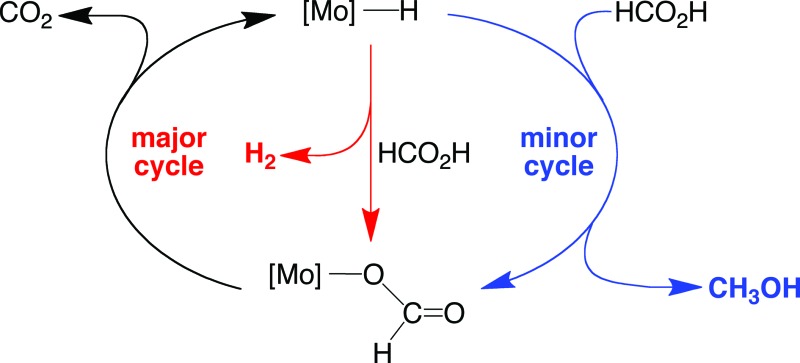
Cyclopentadienyl molybdenum hydride compounds are catalysts for dehydrogenation, disproportionation and transfer hydrogenation reactions of formic acid, in which the latter provides a means to reduce aldehydes and ketones.

## Introduction

The growing demand for energy requires the development of alternative energy sources that are more sustainable than fossil fuels and have a reduced impact on the environment. As such, both the sun and the wind are considered to be major candidates for the production of renewable energy.^1^ However, since energy production from these sources would vary with location and time, it is essential to couple the production of energy with efficient methods for storage and transportation.^2^ In this regard, an attractive energy storage medium is provided by hydrogen,^3^ which can, for example, be consumed in a fuel cell with only water as a byproduct. Unfortunately, a problem with using hydrogen in this manner is that present storage and transportation techniques are inadequate.^4^ For example, not only does storing liquid hydrogen present a safety risk, considerable energy is also required to liquefy the hydrogen and maintain it in this form.^2*c*^

In view of these issues, efforts have focused on the use of physisorption and chemical methods to provide hydrogen on demand.^5^ Of these approaches, the use of formic acid as a chemical medium for storing H_2_ has garnered much attention.^2,6,7^ Specifically, although formic acid contains only 4.4% hydrogen by mass, it is an attractive storage medium because (i) it is a liquid at room temperature and is therefore easy to handle and transport, (ii) it is commercially available on a large scale^8,9^ and (iii) the byproduct of H_2_ release is carbon dioxide which, in principle, can be trapped^10^ and either recycled^11,12^ or used as a C_1_ source for other chemicals.^12^ With respect to achieving the release of H_2_ from formic acid, much research has been directed towards the discovery of both heterogeneous^13^ and homogeneous^14–22^ catalyst systems. However, since most of these catalyst systems feature precious metals (*e.g.* Pd, Pt, Au), an important current thrust is the discovery of catalysts that utilize earth abundant nonprecious metals.^19–22^ Therefore, we report here a series of molybdenum compounds that are not only effective for the catalytic release of H_2_ from formic acid, but are also capable of (i) forming methanol *via* disproportionation of formic acid and (ii) using formic acid as a reagent for the transfer hydrogenation of aldehydes and ketones.

## Results and discussion

**Scheme 1:**
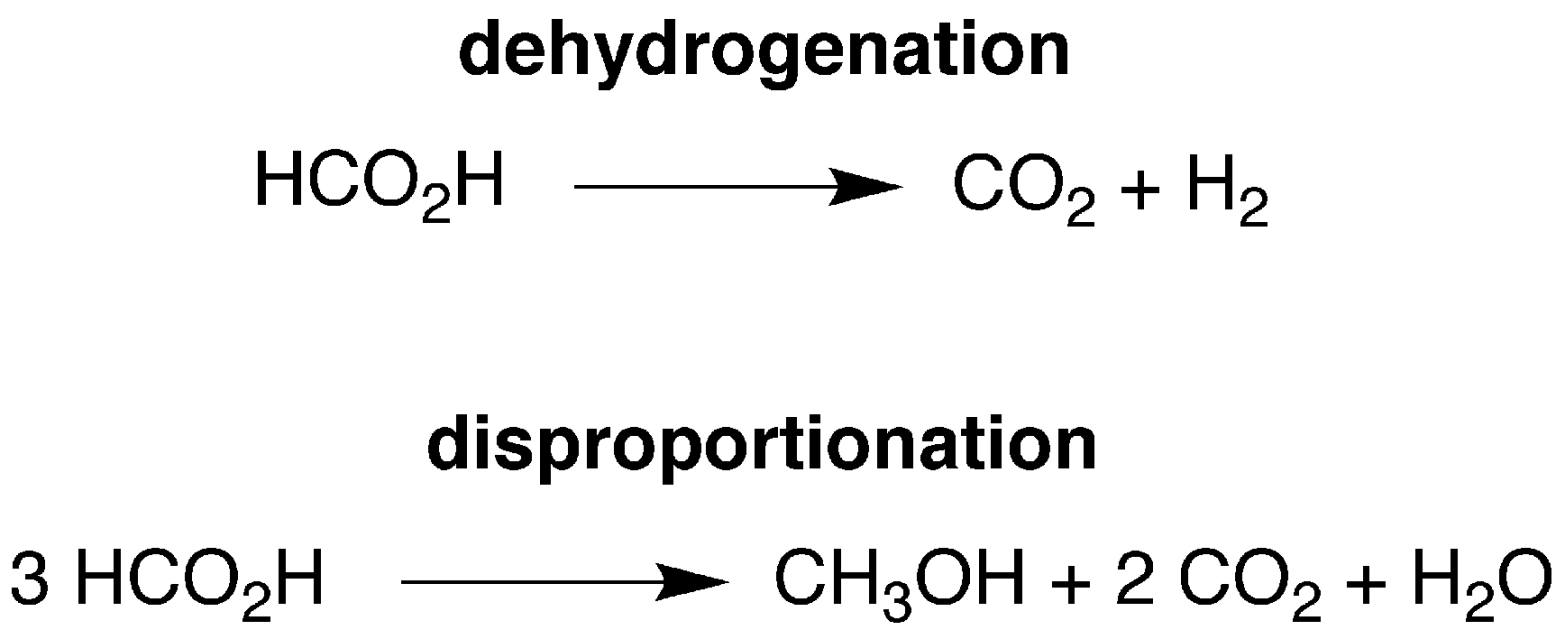
Dehydrogenation and disproportionation of HCO_2_H.

We have previously described the first example of a molybdenum compound, namely Cp*Mo(PMe_3_)_2_(CO)H,^20,23^ to serve as a catalyst for the dehydrogenation (decarboxylation) of formic acid (Scheme 1).^24^ In order to understand the factors that influence dehydrogenation, we sought to extend the investigation to the series of compounds, CpMo(PMe_3_)_3–*x*_(CO)_*x*_H and Cp*Mo(PMe_3_)_3–*x*_(CO)_*x*_H (*x* = 0, 1, 2 or 3), in which the substitution of (i) PMe_3_ ligands by CO and (ii) Cp* ligands by Cp would be expected to exert significant electronic and structural effects, as illustrated by the fact that the oxidation potentials of the CpMo(PMe_3_)_3–*x*_(CO)_*x*_H/[CpMo(PMe_3_)_3–*x*_(CO)_*x*_H]^+^ couples span a range of *ca.* 2 V.^25^

Indeed, comparison of CpMo(PMe_3_)_3_H,^26^ CpMo(PMe_3_)_2_(CO)H,^27^ CpMo(PMe_3_)(CO)_2_H^27,28^ and CpMo(CO)_3_H^29^ indicates that substitution of a CO ligand by PMe_3_ has a profound effect on the rate of decarboxylation and release of H_2_ (Table 1).^30^ For example, while the reaction catalyzed by the tricarbonyl derivative, CpMo(CO)_3_H, requires temperatures of 100 °C in order to proceed efficiently in benzene, the bis(phosphine) derivative, CpMo(PMe_3_)_2_(CO)H, is an effective catalyst at room temperature.^31^ Most interestingly, the trend is not monotonic, such that the tris(phosphine) derivative CpMo(PMe_3_)_3_H exhibits no significant catalytic activity at room temperature. Thus, of the series of compounds CpMo(PMe_3_)_3–*x*_(CO)_*x*_H, the bis(phosphine) derivative, CpMo(PMe_3_)_2_(CO)H, has the greatest activity.

**Table 1:** Turnover frequencies (h^–1^) for dehydrogenation of formic acid by Cp^R^Mo(PMe_3_)_3–*x*_(CO)_*x*_H at 100 °C^*a*^

	Cp	Cp*
Cp^R^Mo(PMe_3_)_2_(CO)H	31	54
Cp^R^Mo(PMe_3_)(CO)_2_H	0.64	1.2
Cp^R^Mo(CO)_3_H	0.67	0.33

^*a*^[Cp^R^Mo(PMe_3_)_3–*x*_(CO)_*x*_H] = 0.016 M, [HCO_2_H]_initial_ = 0.39 M, C_6_D_6_, values at 50% conversion.

**Scheme 2:**
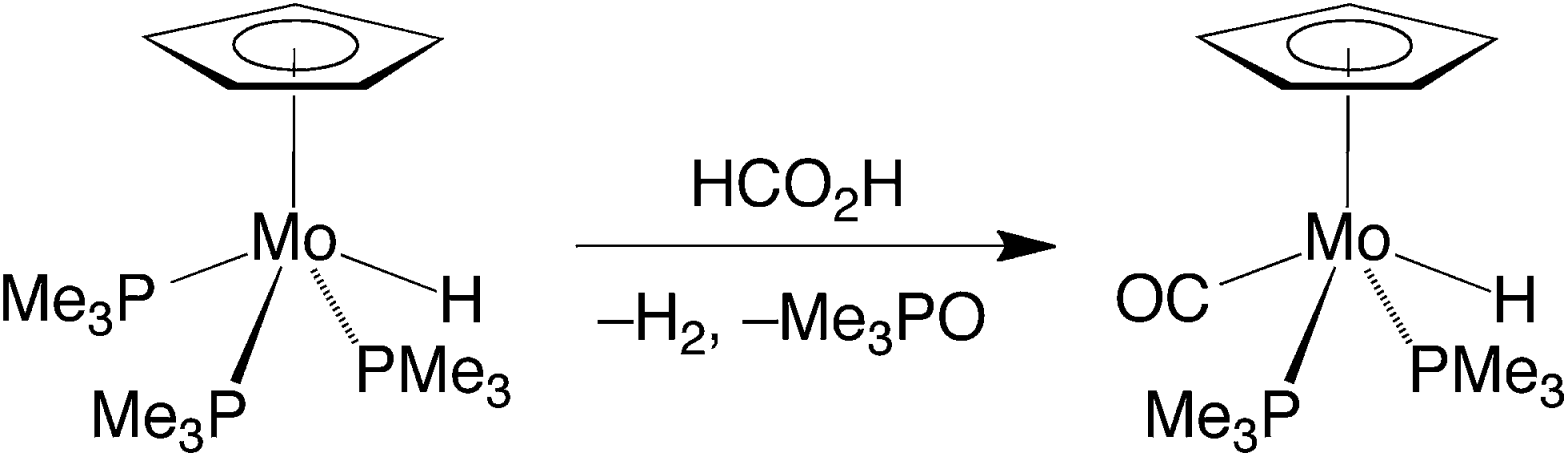
Conversion of CpMo(PMe_3_)_3_H to CpMo(PMe_3_)_2_(CO)H by formic acid.

Although CpMo(PMe_3_)_3_H exhibits little activity at room temperature, it does, nevertheless, induce the decarboxylation of formic acid at elevated temperatures. However, it is evident that CpMo(PMe_3_)_3_H is not necessarily the catalytically active species because it is converted to the monocarbonyl compound CpMo(PMe_3_)_2_(CO)H under these conditions (Scheme 2).^32^

The nature of the substituents on the cyclopentadienyl ring also influences the reactivity, with Cp*Mo(PMe_3_)_2_(CO)H being a more active catalyst than CpMo(PMe_3_)_2_(CO)H (Table 1).^31^ The difference is not, however, as pronounced as that achieved by variation of the number of PMe_3_ and CO ligands.

**Scheme 3:**
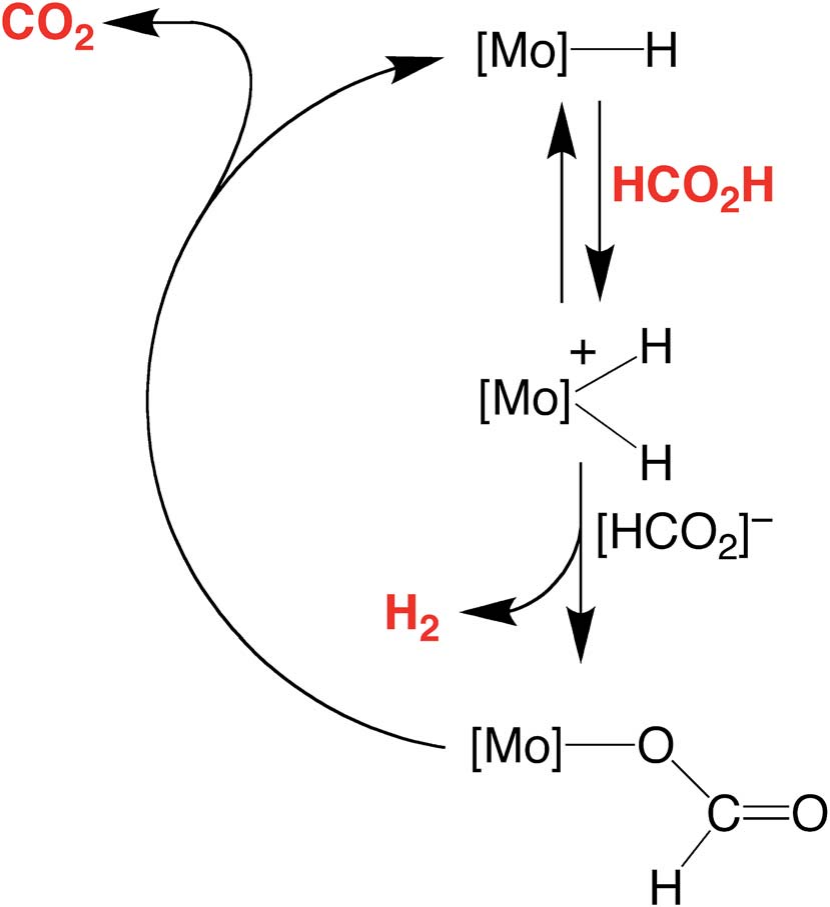
Essential features for the proposed mechanism of dehydrogenation of HCO_2_H by Cp^R^Mo(PMe_3_)_3–*x*_(CO)_*x*_H.

The essential features of the mechanism proposed for the catalytic cycle are illustrated in Scheme 3 and involve two main sequences, namely (i) protonation, elimination of H_2_ and coordination of formate, and (ii) decarboxylation of the formate ligand to regenerate the hydride species. In order to obtain evidence for such a mechanism, the nature of the molybdenum-containing compounds that form upon treatment of CpMo(PMe_3_)_3–*x*_(CO)_*x*_H (*x* = 0, 1, 2, or 3) with formic acid has been probed by NMR spectroscopy.

**Fig. 1:**
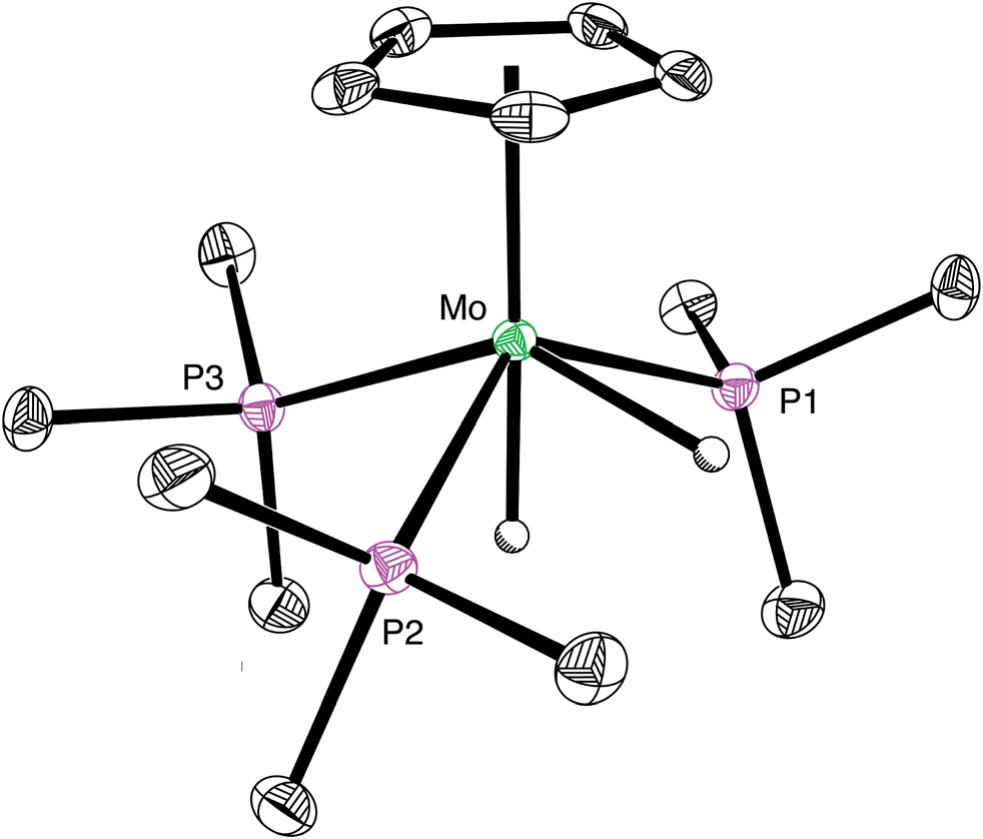
Molecular structure of [CpMo(PMe_3_)_3_H_2_][Cl] (only the cation is shown).

Evidence that CpMo(PMe_3_)_3–*x*_(CO)_*x*_H may be protonated by formic acid is provided by the observation that treatment of CpMo(PMe_3_)_3_H with formic acid results in the formation of [CpMo(PMe_3_)_3_H_2_][HCO_2_], which is characterized by two hydride signals in a 1 : 1 ratio at –2.57 [m, ^2^ *J*_P–H_ = 54, ^2^ *J*_P–H_ = 49 and ^2^ *J*_H–H_ = 8] and –5.74 [m, ^2^ *J*_P–H_ = 46, ^2^ *J*_P–H_ = 8 and ^2^ *J*_H–H_ = 8] in the ^1^H NMR spectrum (CD_3_CN) at 239 K.^33^ Support for the identification of [CpMo(PMe_3_)_3_H_2_][HCO_2_] is provided by previous studies in which [CpMo(PMe_3_)_3_H_2_]^+^ has been generated by the reaction of CpMo(PMe_3_)_3_H with HBF_4_.^25,26a^ Furthermore, we have also structurally characterized the chloride derivative [CpMo(PMe_3_)_3_H_2_][Cl] by X-ray diffraction (Fig. 1), which reinforces its identification as a dihydride rather than a dihydrogen complex.^34^

However, despite the fact that CpMo(PMe_3_)_3_H is readily protonated, it is not, as noted above, an effective catalyst for decarboxylation of formic acid at room temperature. Presumably, the catalytic inactivity of CpMo(PMe_3_)_3_H is a consequence of dissociation of H_2_ from [CpMo(PMe_3_)_3_H_2_][HCO_2_] being non-facile at room temperature. Supporting this suggestion, the tetrafluoroborate derivative, [CpMo(PMe_3_)_3_H_2_][BF_4_], is stable with respect to dissociation of H_2_ at 80 °C.^25^

Evidence for the formation of a protonated species has also been obtained for the reaction of CpMo(PMe_3_)_2_(CO)H with formic acid. Specifically, treatment of CpMo(PMe_3_)_2_(CO)H in *d*_8_-toluene with an excess of formic acid at low temperature (276 K) allows for observation of a protonated species, [CpMo(PMe_3_)_2_(CO)H_2_]^+^, which is characterized by a triplet signal assignable to the hydride ligands at –3.33 ppm [t, ^2^ *J*_P–H_ = 43 Hz] in the ^1^H NMR spectrum.^35,36^ In contrast to [CpMo(PMe_3_)_3_H_2_]^+^, however, [CpMo(PMe_3_)_2_(CO)H_2_]^+^ does not persist at room temperature, dissociating H_2_ and coordinating formate to give CpMo(PMe_3_)_2_(CO)(κ^1^-O_2_CH). The latter compound is also unstable and only exists for a prolonged period in the presence of excess formic acid because it slowly dissociates CO_2_ and regenerates CpMo(PMe_3_)_2_(CO)H.^37^ It is, therefore, evident that both protonation and release of H_2_ is facile in this system, such that decarboxylation is the turnover-limiting step of the catalytic cycle in benzene during the initial stages. However, as the formic acid is consumed, the pseudo first order rate constant for protonation of CpMo(PMe_3_)_2_(CO)H decreases relative to that for decarboxylation of CpMo(PMe_3_)_2_(CO)(κ^1^-O_2_CH), such that CpMo(PMe_3_)_2_(CO)H becomes the resting state during the latter stage of the catalytic transformation.

Unlike CpMo(PMe_3_)_3_H and CpMo(PMe_3_)_2_(CO)H, the dicarbonyl and tricarbonyl compounds, CpMo(PMe_3_)(CO)_2_H and CpMo(CO)_3_H, do not react with formic acid in benzene at room temperature. As such, elevated temperatures are required for efficient catalysis. However, although it is unreactive towards formic acid at room temperature, previous studies have shown that CpMo(PMe_3_)(CO)_2_H reacts with a stronger acid, namely HBF_4_, to release H_2_, with no observation of the dihydride cation, [CpMo(PMe_3_)(CO)_2_H_2_]^+^.^38,39^ Likewise, CpMo(CO)_3_H rapidly eliminates H_2_ upon treatment with TfOH to afford CpMo(CO)_3_OTf, with no observation of [CpMo(CO)_3_H_2_]^+^.^39a,40^ On the basis of these observations, it is evident that the reduced basicity of the metal center resulting from the increased replacement of the PMe_3_ ligands by CO^41,42^ causes protonation to be turnover-limiting.

The above observations clearly indicate that, for the series of CpMo(PMe_3_)_3–*x*_(CO)_*x*_H compounds, the turnover-limiting step depends on the distribution of PMe_3_ and CO ligands. Specifically, (i) the catalytic activities of CpMo(CO)_3_H and CpMo(PMe_3_)(CO)_2_H are limited by their susceptibility to protonation by formic acid, (ii) the activity of CpMo(PMe_3_)_3_H is limited by the inability of [CpMo(PMe_3_)_3_H_2_]^+^ to dissociate H_2_, and (iii) the activity of CpMo(PMe_3_)_2_(CO)H is limited by decarboxylation of the formate intermediate, CpMo(PMe_3_)_2_(CO)(κ^1^-O_2_CH), in the presence of a high concentration of formic acid, but by protonation of CpMo(PMe_3_)_2_(CO)H when the concentration of acid is low.

The different forms of the catalyst resting states provide some insight into the non-monotonic variation in activity upon substituting PMe_3_ for carbonyl ligands. Thus, while CpMo(CO)_3_H has a low activity because the metal center is not susceptible to protonation, CpMo(PMe_3_)_3_H has a low activity because the protonated derivative [CpMo(PMe_3_)_3_H_2_]^+^ is relatively stable with respect to elimination of H_2_. As such, the hybrid carbonyl/phosphine derivatives, Cp^R^Mo(PMe_3_)_2_(CO)H, have the greatest reactivity.

Interestingly, while CO_2_ and H_2_ are the principal products of the catalytic conversion of formic acid by Cp^R^Mo(PMe_3_)_3–*x*_(CO)_*x*_H (R = H, Me), methanol and methyl formate are also observed. The formation of methanol is a consequence of disproportionation of formic acid (Scheme 1), while the methyl formate is a result of subsequent esterification. The observation of this additional pathway is of note in view of the fact that the generation of methanol is a key component of the potential methanol economy,^43^ and yet there are only two reports for the formation of methanol by the homogeneous catalytic disproportionation of formic acid.^15g,17c,44^ Furthermore, both of these reports describe catalysts that contain precious metals, namely iridium^17*c*^ and ruthenium.^15*g*^ As such, the ability to catalyze this transformation by compounds containing the nonprecious metal, molybdenum, is noteworthy. The selectivity for the catalytic formation of methanol and methyl formate relative to decarboxylation by Cp^R^Mo(PMe_3_)_3–*x*_(CO)_*x*_H depends on the nature of the cyclopentadienyl substituents and the number of carbonyl ligands, and is greatest for CpMo(CO)_3_H, which can achieve a selectivity^45^ (21%) that is intermediate between the values for the iridium (12%)^17*c*^ and ruthenium (27%)^15g,46^ systems.

**Scheme 4:**
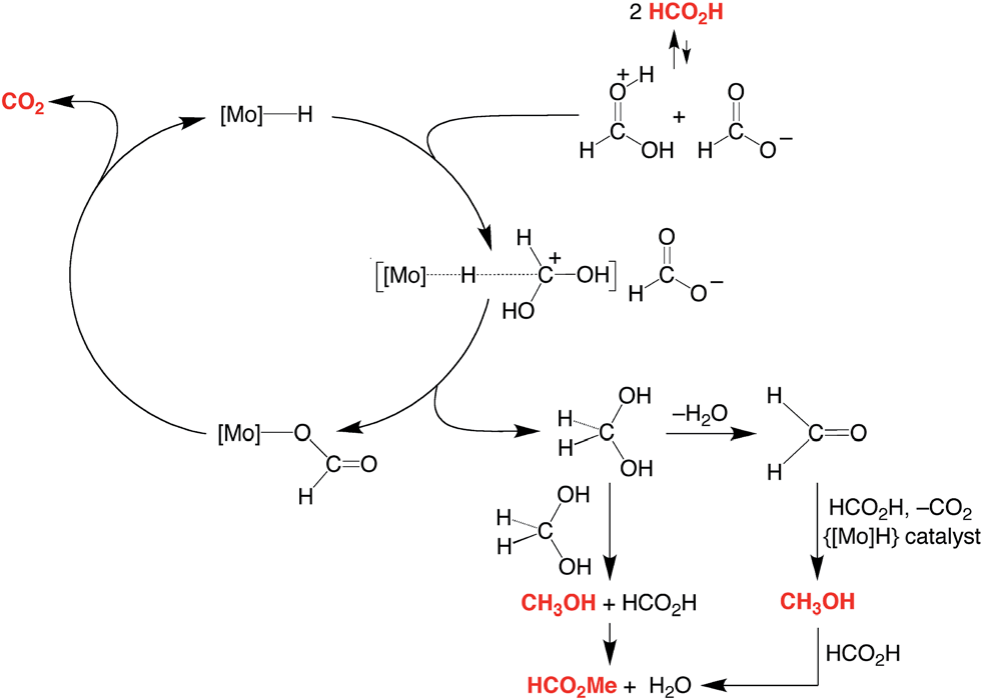
Essential features for the proposed mechanism of disproportionation of HCO_2_H by Cp^R^Mo(PMe_3_)_3–*x*_(CO)_*x*_H.

A simplified mechanism for the formation of methanol is illustrated in Scheme 4 and is based on that previously proposed for the catalytic ionic hydrogenation of ketones.^34,39,47–49^ Thus, it is proposed that Cp^R^Mo(PMe_3_)_3–*x*_(CO)_*x*_H delivers a hydride ligand to the protonated form of formic acid, [HC(OH)_2_]^+^, which is present due to autoionization^50^ or generated incipiently, thereby forming methylene diol. The formation of methanol from CH_2_(OH)_2_ can be achieved in principle by either (i) disproportionation to methanol and formic acid,^51^ or by (ii) dehydration to formaldehyde followed by catalytic ionic hydrogenation akin to that for the first reduction sequence of formic acid (Scheme 4).

Support for a direct proton and hydride transfer, rather than reaction with the H_2_ released *via* decarboxylation, is provided by the observation that performing the reaction under D_2_ (1 atm) results in no deuterium incorporation into the methanol or methyl formate.

**Scheme 5:**
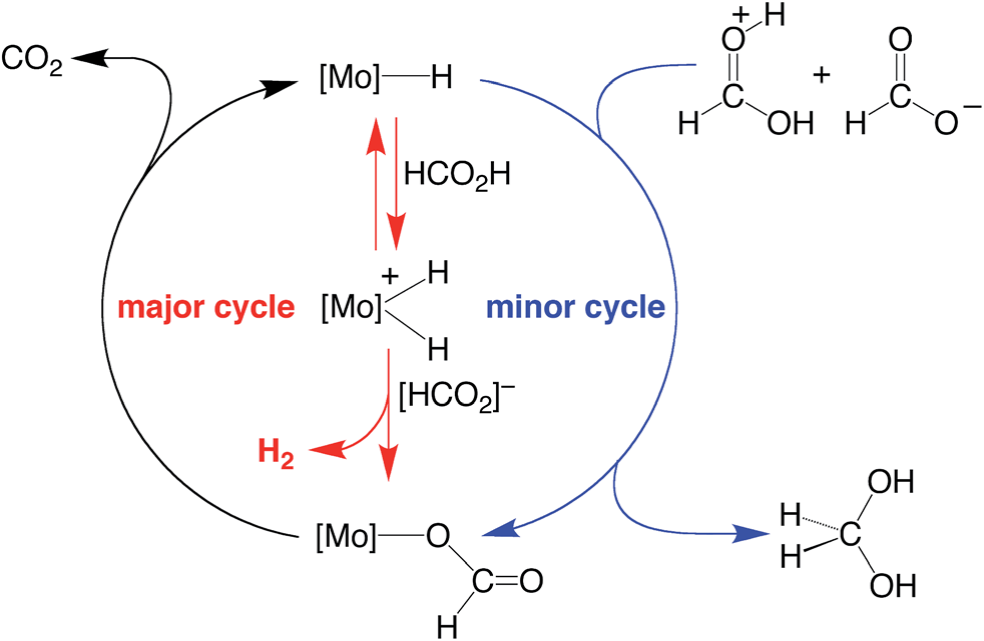
Possible origin of the selectivity of disproportionation *versus* dehydrogenation of formic acid.

On the basis of the mechanisms illustrated in Scheme 3 and Scheme 4 for the dehydrogenation and disproportionation of formic acid, the relative selectivity of the two processes (dehydrogenation *versus* disproportionation) is influenced by the preference of the metal center to transfer a hydride ligand to [HC(OH)_2_]^+^ (which is present in low concentration) relative to its tendency to undergo protonation and liberate H_2_ (Scheme 5). As such, the p*K*_B_ and the hydricity of Cp^R^Mo(PMe_3_)_3–*x*_(CO)_*x*_H are expected to play an important role in influencing the selectivity. In this regard, while such values have been reported for a variety of metal hydride derivatives,^41,52–54^ data for all the compounds described here are not available. Nevertheless, the kinetic hydricity of CpMo(PMe_3_)(CO)_2_H is significantly greater than that of CpMo(CO)_3_H, by a factor of *ca.* 10^4^,^52^ which is in accord with the notion that replacing a CO ligand by PMe_3_ would be expected to promote dissociation of hydride.^55^ However, it is also generally recognized that the basicity of a metal center increases upon replacing a CO ligand by PR_3_ (*vide supra*),^41,42^ and so it is nontrivial to predict *a priori* the relative influence of such substitution on the two pathways.

**Scheme 6:**
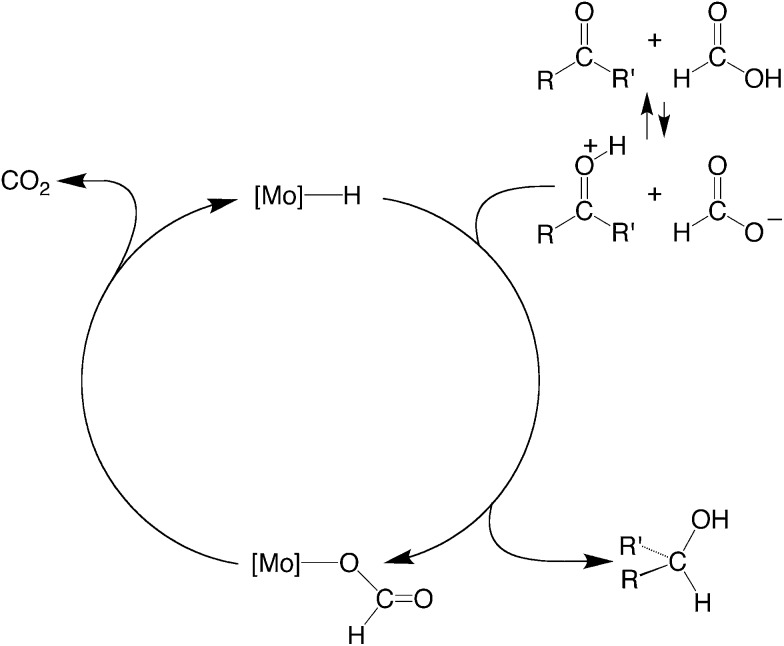
Ionic hydrogenation of carbonyl compounds.

The ability of these molybdenum compounds to catalyze the disproportionation of formic acid prompted the possibility that a system could be developed to use formic acid as a reagent in the transfer hydrogenation of other substrates. In this regard, transfer hydrogenation,^56,57^ often employing isopropanol as the reductant, offers considerable advantages over direct reduction by hydrogen because it obviates the need to use H_2_.^56^ However, disadvantages of using isopropanol are that (i) isopropanol is obtained commercially by the hydration of propene,^58^ and (ii) the reactions are often reversible, such that the isopropanol needs to be used as a solvent to drive the equilibrium.^56^ In contrast, formic acid is available from renewable sources, and the release of CO_2_ as a byproduct renders the reaction effectively irreversible.^56^ However, catalysts that are commonly used to effect transfer hydrogenation using formic acid are largely restricted to the platinum group metals, namely Ru, Rh, Pd and Ir,^59–61^ with few reports employing other metals,^62^ and none employing Mo. It is, therefore, noteworthy that CpMo(CO)_3_H is also capable of effecting transfer hydrogenation of a variety of carbonyl compounds, namely RCHO (R = Me, Pr^i^), RC(O)Me (R = Me, Pr^i^, Bu^t^, Ph) and Ph_2_CO, using formic acid as the reductant,^63^ as illustrated in Scheme 6. For example, Ph_2_CO is reduced by HCO_2_H to Ph_2_CH(OH) with a selectivity of 29% relative to decarboxylation and disproportionation, while Pr^i^CHO is reduced to Bu^i^OH and HCO_2_Bu^i^ (3 : 1) with a selectivity of 32%.^64–67^

## Conclusions

In summary, the cyclopentadienyl molybdenum hydride compounds, Cp^R^Mo(PMe_3_)_3–*x*_(CO)_*x*_H (Cp^R^ = Cp, Cp*; *x* = 0, 1, 2 or 3), are catalysts for the dehydrogenation of formic acid. The activity of these catalysts is not a monotonic function of the number of CO or PMe_3_ ligands and is greatest for Cp^R^Mo(PMe_3_)_2_(CO)H. Specifically, while CpMo(CO)_3_H has a low activity because the metal center is not susceptible towards protonation, CpMo(PMe_3_)_3_H has a low activity because the protonated derivative [CpMo(PMe_3_)_3_H_2_]^+^ is relatively stable with respect to elimination of H_2_. As such, the hybrid carbonyl/phosphine derivatives, Cp^R^Mo(PMe_3_)_2_(CO)H, have the greatest reactivity. In addition to catalyzing the dehydrogenation of formic acid, Cp^R^Mo(PMe_3_)_3–*x*_(CO)_*x*_H also catalyzes its disproportionation to methanol and CO_2_ *via* a transfer hydrogenation reaction. Similarly, CpMo(CO)_3_H also catalyzes the reduction of aldehydes and ketones by formic acid *via* a mechanism that involves ionic hydrogenation. These investigations demonstrate that molybdenum hydride compounds have much potential with respect to transfer hydrogenation reactions involving formic acid.
