# Supplementary material for: Dehydrogenation, disproportionation and transfer hydrogenation reactions of formic acid catalyzed by molybdenum hydride compounds
Source: Chem Sci. 2015 Jan 14;6(3):1859–65. doi: 10.1039/c4sc03128h (PMC5649328; doi:10.1039/c4sc03128h)
Supplement: Supplementary file 1 [file SC-006-C4SC03128H-s001.pdf]

Electronic Supplementary Material (ESI) for Chemical Science.
This journal is © The Royal Society of Chemistry 2014

SUPPORTING INFORMATION

Dehydrogenation, Disproportionation and Transfer Hydrogenation Reactions of Formic Acid Catalyzed by Molybdenum Hydride Compounds

Michelle C. Neary and Gerard Parkin,*

Department of Chemistry,

Columbia University,

New York, New York 10027, USA.

E-mail: parkin@columbia.edu

Received xxxx xx, 2014.

General Considerations

All manipulations were performed using a combination of glovebox, high vacuum and Schlenk techniques under a nitrogen or argon atmosphere.[1] Solvents were purified and degassed by standard procedures. NMR spectra were measured on Bruker 300 DRX, Bruker 400 DRX, and Bruker Avance 500 DMX spectrometers. ^{1}H NMR spectra are reported in ppm relative to $SiMe_4$ (δ = 0) and were referenced internally with respect to the protio solvent impurity (δ = 7.16 for C_6D_5H, δ = 2.08 for C_7D_7H, δ = 1.94 for CD_2HCN and δ = 1.72 for d_8-THF).[2] When required for quantitative integration, ^{1}H NMR spectra were acquired with an extended d1 of 60 s and mesitylene was used as an internal standard. ^{13}C NMR spectra are reported in ppm relative to $SiMe_4$ (δ = 0) and were referenced internally with respect to the solvent (δ = 128.06 for C_6D_6 and δ = 118.26 for CD_3CN).[2] ^{31}P NMR chemical shifts are reported in ppm relative to 85% H_3PO_4 (δ = 0) and were referenced externally using $(MeO)_3P$ (δ = 141.0).[3] Coupling constants are reported in hertz. Infrared spectra were recorded on a Nicolet iS10 spectrometer and a Perkin Elmer Spectrum Two spectrometer, and are reported in reciprocal centimeters. $CpMo(CO)_3H$,[4] $CpMo(PMe_3)(CO)_2H$,[5] $CpMo(PMe_3)_2(CO)H$,[5b] $CpMo(PMe_3)_3H$,[6] $Cp^*Mo(CO)_3H$,[7] $Cp^*Mo(PMe_3)(CO)_2H$[8] and $Cp^*Mo(PMe_3)_2(CO)H$[5b,9] have been reported and were prepared by the literature methods or modifications thereof, as described below. Other chemicals were obtained from Sigma-Aldrich [formic acid, mesitylene, 1.0 M HCl in Et_2O, $Pr^iC(O)Me$, $Bu^tC(O)Me$, PhC(O)Me and Ph_2CO], Acros Organics [MeCHO and Pr^iCHO], Fisher [MeC(O)Me], Alfa Aesar [TfOH], Cambridge Isotope Laboratories [$H^{13}CO_2H$ and D_2] and TechAir [H_2], and used as supplied.

X-ray Structure Determination

X-ray diffraction data were collected on a Bruker Apex II diffractometer. Crystal data, data collection and refinement parameters are summarized in Table 5. The structure was solved using direct methods and standard difference map techniques, and was refined by full-matrix least-squares procedures on F^2 with SHELXTL (Version 2008/4).[10]

Synthesis of $CpMo(CO)_3H$

$CpMo(CO)_3H$ was prepared by using a modified literature method.[4] A suspension of $Mo(CO)_6$ (5.00 g, 18.9 mmol) and NaCp (2.00 g, 22.7 mmol) in THF (*ca.* 60 mL) was refluxed overnight, thereby resulting in the formation of a clear brown solution. The volatile components were removed *in vacuo* to give a tan solid, which was treated with a degassed aqueous solution of NaOH (25 mL of 0.50 M), resulting in a cloudy brown suspension. The mixture was filtered and the filtrate was treated with degassed aqueous acetic acid (20 mL of 2.0 M), resulting in the immediate formation of a pale yellow precipitate, which was isolated by filtration and dried *in vacuo* to give $CpMo(CO)_3H$ as a pink microcrystalline solid (4.24 g, 91% yield). ^{1}H NMR (C_6D_6): -5.47 [s, 1H, $CpMo(CO)_3\underline{H}$], 4.56 [s, 5H, $(C_5\underline{H}_5)Mo(CO)_3H$].[11]

Synthesis of $CpMo(PMe_3)(CO)_2H$

$CpMo(PMe_3)(CO)_2H$ was prepared by using a modified literature method.[5] A solution of $CpMo(CO)_3H$ (120 mg, 0.49 mmol) in benzene (*ca.* 1 mL) was cooled to –196˚C and treated with PMe_3 (0.50 mL, 4.8 mmol) *via* vapor transfer. The mixture was allowed to warm to room temperature, resulting in a bright yellow solution. The solution was filtered to remove a small amount of tan precipitate, and the filtrate was lyophilized to give $CpMo(PMe_3)(CO)_2H$ as a yellow powder (97 mg, 68% yield). Anal. calcd. for $CpMo(PMe_3)(CO)_2H$: C, 40.8%; H, 5.1%. Found: C, 40.7%; H, 4.9%. ^{1}H NMR (C_6D_6): -5.89 [br, 1H, $CpMo(PMe_3)(CO)_2\underline{H}$], 1.02 [d, 9H, $CpMo(P(C\underline{H}_3)_3)(CO)_2H$, $^2J_{P-H} = 9$], 4.77 [s, 5H, $(C_5\underline{H}_5)Mo(PMe_3)(CO)_2H$].

Synthesis of $CpMo(PMe_3)_2(CO)H$

$CpMo(PMe_3)_2(CO)H$ was prepared by using a modified literature method.[5b] A solution of $CpMo(CO)_3H$ (100 mg, 0.406 mmol) in benzene (*ca.* 1 mL) was cooled to -196˚C and treated with PMe_3 (0.15 mL, 1.5 mmol) *via* vapor transfer. The mixture

was allowed to warm to room temperature, resulting in a bright yellow solution and the formation of $CpMo(PMe_3)(CO)_2H$. The solution was degassed *via* one freeze-pump-thaw cycle to remove excess CO and was then irradiated (350 nm) for *ca.* 30 hours, with occasional degassing *via* freeze-pump-thaw cycles. The mixture was filtered, and the filtrate was lyophilized to give $CpMo(PMe_3)_2(CO)H$ as a yellow powder (41 mg, 29% yield). Anal. calcd. for $CpMo(PMe_3)_2(CO)H$: C, 42.1%; H, 7.1%. Found: C, 41.8%; H, 6.7%. ^1H NMR (C_6D_6): -6.93 [t, 1H, $CpMo(PMe_3)_2(CO)\underline{H}$, $^2J_{P\text{-}H} = 74$], 1.28 [d, 18H, $CpMo(P(C\underline{H}_3)_3)_2(CO)H$, $^2J_{P\text{-}H} = 9$], 4.87 [s, 5H, $(C_5\underline{H}_5)Mo(PMe_3)_2(CO)H$]. $^{13}C\{^1H\}$ NMR (C_6D_6): 26.20 [d, $CpMo(P(\underline{C}H_3)_3)_2(CO)H$, $^1J_{P\text{-}C} = 30$], 87.53 [s, $(\underline{C}_5H_5)Mo(PMe_3)_2(CO)H$], 248.53 [t, $CpMo(PMe_3)_2(\underline{C}O)H$, $^2J_{P\text{-}C} = 27$]. $^{31}P\{^1H\}$ NMR (C_6D_6): 32.94 [s, $CpMo(\underline{P}Me_3)_2(CO)H$].

Synthesis of $CpMo(PMe_3)_3H$

$CpMo(PMe_3)_3H$ was prepared by using a modified literature procedure.[6a] A solution of freshly distilled CpH (20 μL, 0.24 mmol) in benzene (0.7 mL) was treated with $Mo(PMe_3)_6$ (50 mg, 0.091 mmol) and heated at 80˚C for four hours in a sealed tube. After this period, the dark yellow suspension was lyophilized. The residue obtained was extracted into benzene (*ca*. 0.7 mL), and the extract was lyophilized to give $CpMo(PMe_3)_3H$ as a yellow powder (24 mg, 69%). ^1H NMR (C_6D_6): -8.36 [q, 1H, $CpMo(PMe_3)_3\underline{H}$, $^2J_{P\text{-}H} = 52$], 1.29 [filled in d, 27H, $CpMo(P(C\underline{H}_3)_3)_3H$, $^2J_{P\text{-}H} = 6$], 4.47 [s, 5H, $(C_5\underline{H}_5)Mo(PMe_3)_3H$].[6a]

Synthesis of $Cp^*Mo(PMe_3)_2(CO)H$

$Cp^*Mo(PMe_3)_2(CO)H$ was prepared by using a modified literature procedure.[5b] A solution of $Cp^*Mo(CO)_3H$ (32 mg, 0.10 mmol) in benzene (*ca.* 1 mL) was cooled to -196˚C and treated with PMe_3 (0.10 mL, 1.0 mmol) *via* vapor transfer. The mixture was allowed to warm to room temperature and degassed *via* a freeze-pump-thaw cycle. The mixture was irradiated (350 nm) for *ca.* 25 hours, with occasional degassing *via*

freeze-pump-thaw cycles. After this period, the mixture was filtered to remove a tan precipitate, and the filtrate was lyophilized. The residue obtained was triturated with pentane and dried *in vacuo* to give $Cp^*Mo(PMe_3)_2(CO)H$ as a brown solid (28 mg, 67% yield). ^1H NMR(C_6D_6): -6.84 [t, 1H, $Cp^*Mo(PMe_3)_2(CO)\underline{H}$, $^2J_{P\text{-}H} = 78$], 1.32 [d, 18H, $Cp^*Mo(P(C\underline{H}_3)_3)_2(CO)H$, $^2J_{P\text{-}H} = 8$], 1.91 [s, 15H, $(C_5(C\underline{H}_3)_5)Mo(PMe_3)_2(CO)H$].

Reactivity of $CpMo(PMe_3)_3H$ towards Formic Acid

1. Synthesis of $[CpMo(PMe_3)_3H_2][HCO_2]$ in Pentane

Formic acid (14 μL, 0.37 mmol) was added to a yellow solution of $CpMo(PMe_3)_3H$ (14 mg, 0.36 mmol) in pentane (*ca.* 0.7 mL). The solution immediately turned a pale tan color, and the solvent was removed *in vacuo*. The resulting orange residue was triturated with THF (*ca.* 0.7 mL), dried *in vacuo* overnight and rinsed with pentane (*ca.* 0.7 mL). Further drying *in vacuo* gave $[CpMo(PMe_3)_3H_2][HCO_2]$ as a yellow powder (9 mg, 56%). Anal. calcd. for $[CpMo(PMe_3)_3H_2][HCO_2]$: C, 41.3%; H, 8.1%. Found: C, 39.9%; H, 8.1%. ^1H NMR (CD_3CN): –3.94 [br, 2H, $[CpMo(PMe_3)_3\underline{H_2}][HCO_2]$], 1.48 [d, 27H, $[CpMo(P(C\underline{H}_3)_3)_3H_2][HCO_2]$, $^2J_{P\text{-}H} = 9$], 4.82 [m, 5H, $[(C_5\underline{H}_5)Mo(PMe_3)_3H_2][HCO_2]$], 8.45 [s, 1H, $[CpMo(PMe_3)_3H_2][\underline{H}CO_2]$]. ^1H NMR (CD_3CN, 239K):[12] –5.74 [m, 1H, $[CpMo(PMe_3)_3\underline{H_2}][HCO_2]$, $^2J_{P\text{–}H} = 46$, $^2J_{P\text{–}H} = 8$ and $^2J_{H\text{–}H} = 8$], –2.57 [m, 1H, $[CpMo(PMe_3)_3\underline{H_2}][HCO_2]$, $^2J_{P\text{–}H} = 54$, $^2J_{P\text{–}H} = 49$ and $^2J_{H\text{–}H} = 8$], 1.43 [d, 27H, $[CpMo(P(C\underline{H}_3)_3)H_2][HCO_2]$, $^2J_{P\text{-}H} = 8$], 4.79 [s, 5H, $[(C_5\underline{H}_5)Mo(PMe_3)_3H_2][HCO_2]$], 8.42 [s, 1H, $[CpMo(PMe_3)_3H_2][\underline{H}CO_2]$] (coupling constants were determined by $^1H\{selec\text{-}^1H\}$ and $^1H\{selec\text{-}^{31}P\}$ decoupling experiments). $^{31}P\{^1H\}$ NMR (CD_3CN): 8.96 (br). $^{31}P\{^1H\}$ NMR (CD_3CN, 239K): 3.51 [t, 1P, $[CpMo(\underline{P}Me_3)_3H_2][HCO_2]$, $^2J_{P\text{-}P} = 25$], 10.11 [d, 2P $[CpMo(\underline{P}Me_3)_3H_2][HCO_2]$, $^2J_{P\text{-}P} = 25$]. IR (cm^{-1}): 3420 (w), 3091 (w), 2975 (w), 2910 (w), 2812 (w), 1900 (w), 1796 (w), 1659 (m), 1601 (m), 1424 (m), 1395 (m), 1307 (m), 1287 (m), 1195 (m), 1104 (m), 1001 (m), 940 (s), 851 (m), 816 (m), 749 (m), 719 (m), 671 (s), 606 (m), 562 (m).

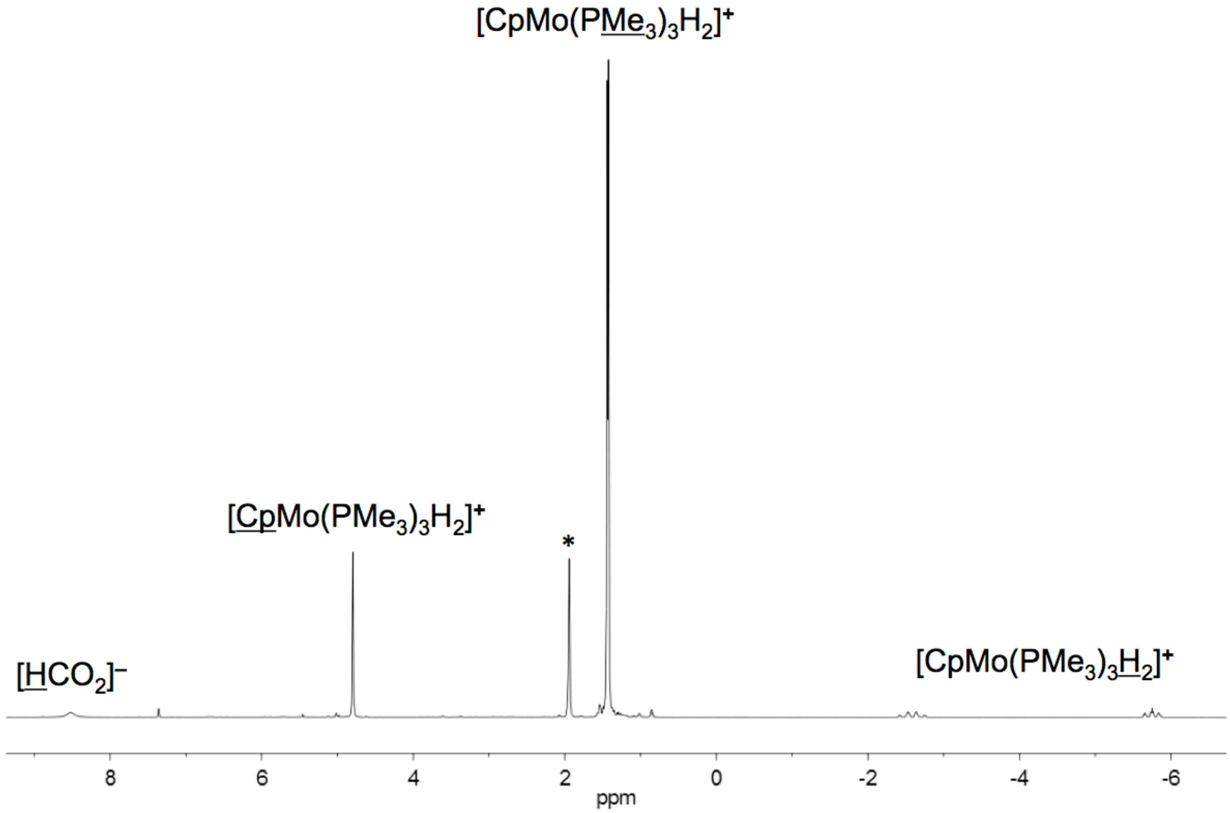

Figure 1. ¹H NMR spectrum for $[CpMo(PMe_3)_3H_2][HCO_2]$ in CD_3CN (*) at 239K

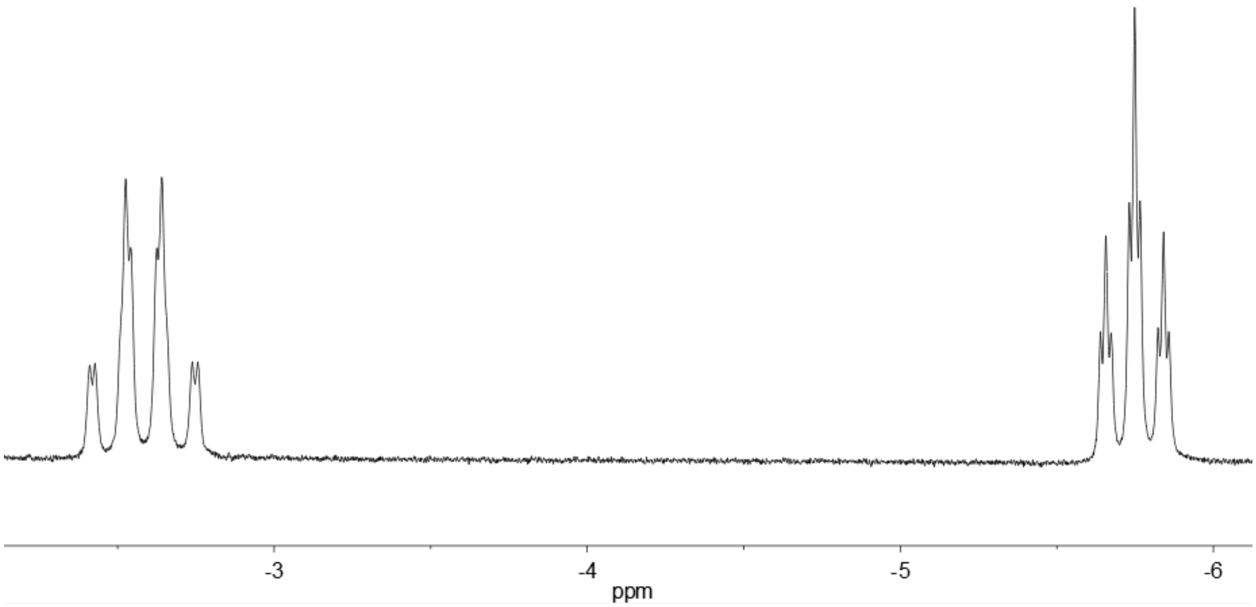

Figure 2. Hydride region of the ¹H NMR spectrum for $[CpMo(PMe_3)_3H_2][HCO_2]$ in CD_3CN at 239K

2. Reactivity of $CpMo(PMe_3)_3H$ towards Formic Acid in Benzene

Formic acid (9.4 μL, 0.25 mmol) was added to a solution of $CpMo(PMe_3)_3H$ (3.9 mg, 0.010 mmol) in C_6D_6 (0.64 mL) containing mesitylene (3.0 μL, 0.022 mmol) as an internal standard, and the reaction was monitored by ^1H NMR spectroscopy. The formation of $[CpMo(PMe_3)_3H_2][HCO_2]$, *inter alia*, was observed immediately, but there was little conversion of the formic acid to H_2, methyl formate, and methanol over a period of several days at room temperature. Therefore, the mixture was heated to 100°C, at which point $[CpMo(PMe_3)_3H_2][HCO_2]$ converted rapidly to $CpMo(PMe_3)_2(CO)H$ and $CpMo(PMe_3)_2(CO)(\kappa^1\text{-}O_2CH)$, and the formic acid converted to H_2, methyl formate, and methanol.

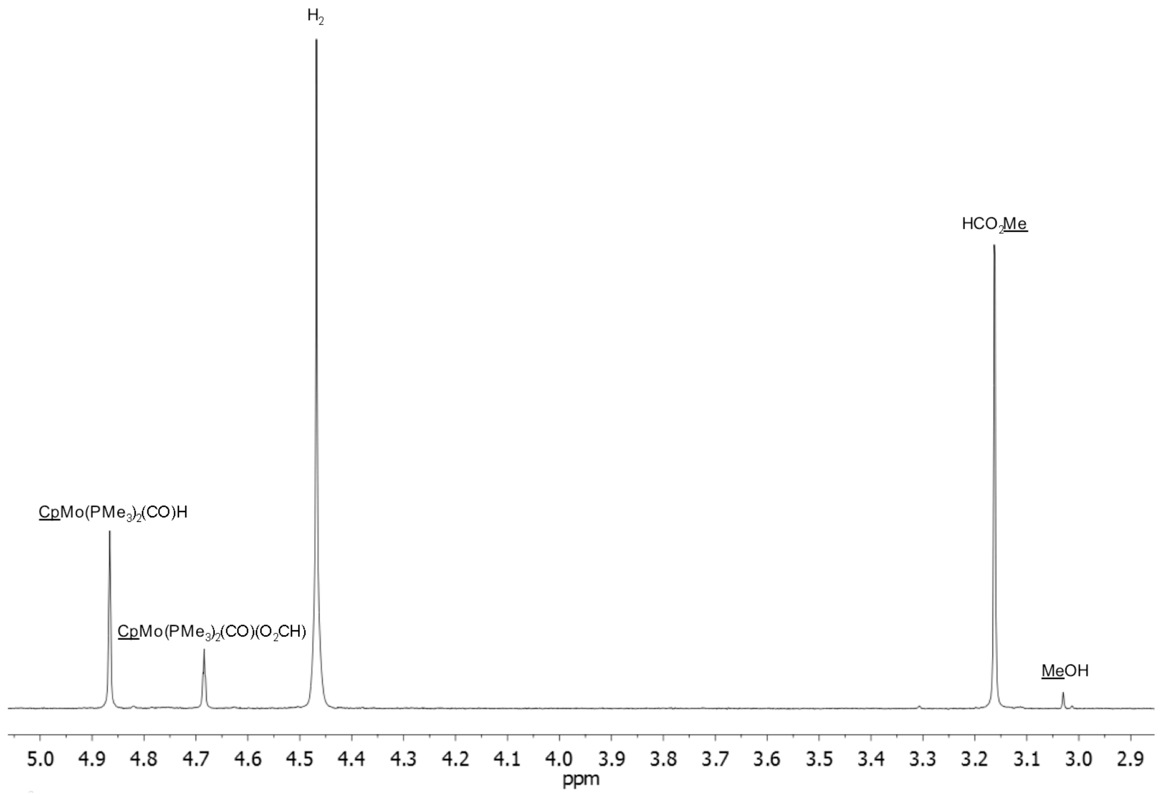

Figure 3. ^1H NMR spectrum of a solution of $CpMo(PMe_3)_3H$ in C_6D_6 in the presence of formic acid after heating at 100°C for 1 day, illustrating the conversion to $CpMo(PMe_3)_2(CO)H$ and $CpMo(PMe_3)_2(CO)(\kappa^1\text{-}O_2CH)$ and the catalytic formation of H_2, HCO_2Me and MeOH.

Synthesis of $[CpMo(PMe_3)_3H_2][Cl]$

A vial containing a solution of $CpMo(PMe_3)_3H$ (7.0 mg, 0.018 mmol) in THF (*ca.* 0.7 mL) was placed into a Schlenk tube. A solution of HCl in Et_2O (0.4 mL of 1.0 M, 0.4 mmol) was injected into the Schlenk tube and allowed to diffuse into the vial overnight. The volatile components of the reaction mixture were removed *in vacuo*, and the brown solid was washed with THF (3 × 0.7 mL). The product was extracted into CH_3CN (*ca.* 0.7 mL), filtered and dried *in vacuo*. The residue was triturated with pentane (*ca.* 0.7 mL) and dried *in vacuo* to give $[CpMo(PMe_3)_3H_2][Cl]$ as a tan powder (3.0 mg, 39% yield). Orange crystals suitable for X-ray diffraction were obtained from a separate reaction in which HCl was allowed to diffuse into a solution of $CpMo(PMe_3)_3H$ (12 mg, 0.031 mmol) in THF (*ca.* 0.7 mL). ^{1}H NMR (CD_3CN): –3.98 [br, 2H, $[CpMo(PMe_3)_3\underline{H}_2][Cl]$], 1.49 [d, 27H, $[CpMo(P(C\underline{H}_3)_3)_3H_2]Cl$, $^{2}J_{P\text{-}H} = 8$], 4.83 [s, 5H, $[(C_5\underline{H}_5)Mo(PMe_3)_3H_2][Cl]$]. $^{13}C\{^{1}H\}$ NMR (CD_3CN): 25.88 [d, $[CpMo(P(\underline{C}H_3)_3)_3H_2][Cl]$, $^{1}J_{P\text{-}C} = 31$], 86.75 [s, $[(\underline{C}_5H_5)Mo(PMe_3)_3H_2][Cl]$]. $^{31}P\{^{1}H\}$ NMR (CD_3CN): 9.04 [br, $[CpMo(\underline{P}Me_3)_3H_2][Cl]$]. IR ($cm^{-1}$): 3371 (vw), 3051 (vw), 3026 (vw), 2967 (w), 2905 (w), 2812 (vw), 1866 (w), 1794 (w), 1716 (vw), 1644 (w), 1424 (m), 1366 (vw), 1306 (w), 1286 (m), 1201 (vw), 1106 (vw), 1016 (w), 939 (s), 835 (m), 719 (m), 671 (s).

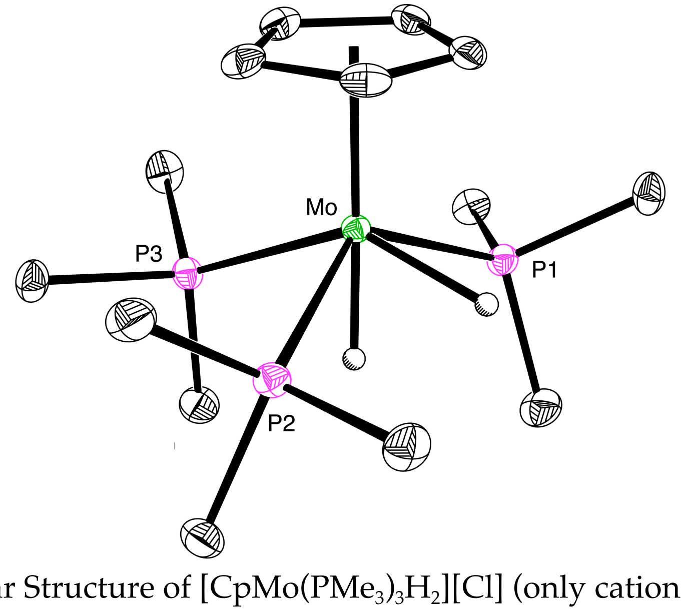

Figure 4. Molecular Structure of $[CpMo(PMe_3)_3H_2][Cl]$ (only cation shown).

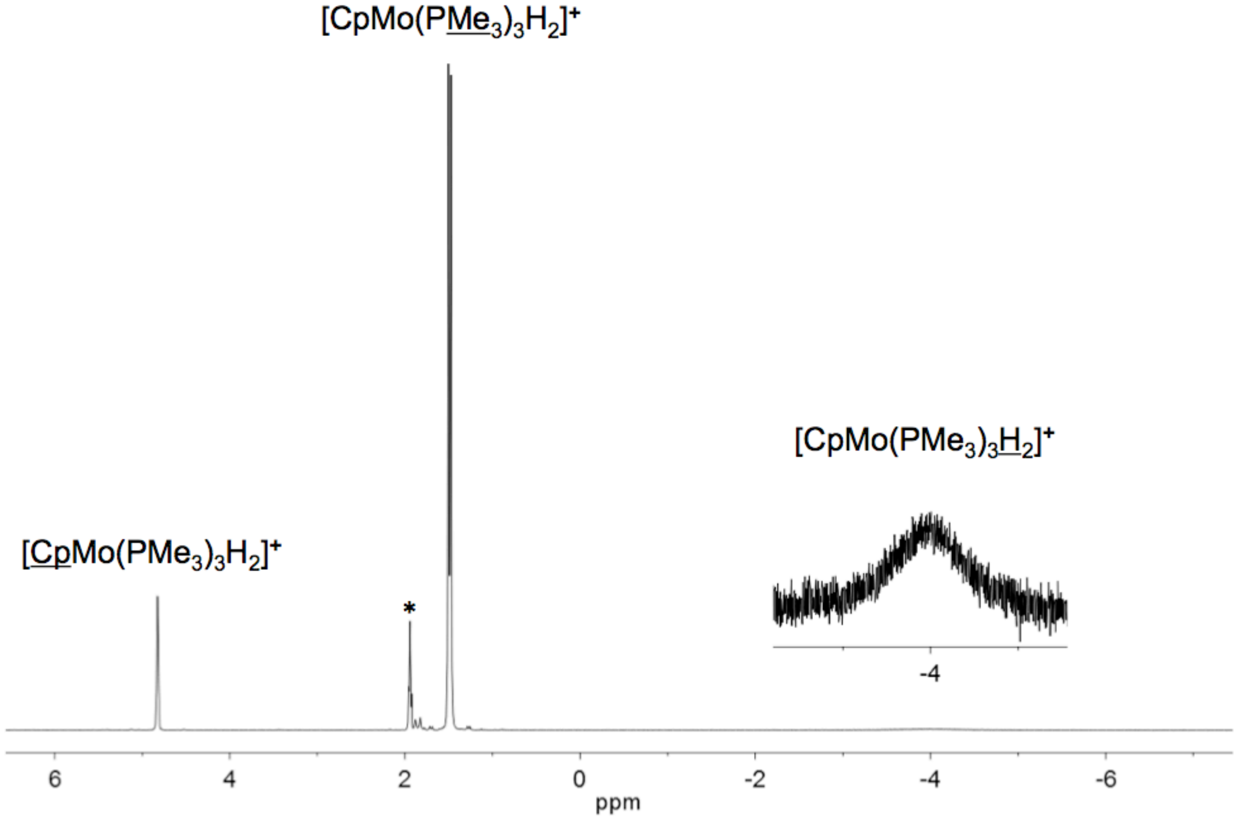

Figure 5. ^{1}H NMR spectrum of [CpMo(PMe$_3$)$_3$H$_2$][Cl] in CD$_3$CN (*). The hydride signal is broad at room temperature, in accord with that of [CpMo(PMe$_3$)$_3$][BF$_4$].[6]

Reactivity of CpMo(PMe$_3$)$_2$(CO)H towards Formic Acid

1. Formation of CpMo(PMe$_3$)$_2$(CO)(κ^1-O$_2$CH) in Benzene

A yellow solution of CpMo(PMe$_3$)$_2$(CO)H (3 mg, 0.009 mmol) in C$_6$D$_6$ (*ca.* 0.7 mL) was treated with an excess of formic acid (0.4 mmol). The solution immediately turned darker yellow, indicating complete conversion to CpMo(PMe$_3$)$_2$(CO)(κ^1-O$_2$CH), as identified by ^{1}H, ^{13}C and ^{31}P NMR spectroscopies. ^{1}H NMR (C$_6$D$_6$): 1.00 [filled in d, 18H, CpMo(CO)(P(C$\underline{H}_3$)$_3$)$_2$(κ^1-O$_2$CH), $^2J_{P\text{-}H}$ = 9], 4.73 [s, 5H, (C$_5\underline{H}_5$)Mo(PMe$_3$)$_2$(CO)(κ^1-O$_2$CH)], 8.30 [t, 1H, CpMo(PMe$_3$)$_2$(CO)(κ^1-O$_2$C$\underline{H}$), $^4J_{P\text{-}H}$ = 2]. Note: the chemical shift of (O$_2$C$\underline{H}$) varies slightly (*ca.* δ 8.3 – 8.6) with concentration. ^{13}C{^{1}H} NMR (C$_6$D$_6$): 16.59 [m, CpMo(CO)(P($\underline{C}$H$_3$)$_3$)$_2$(κ^1-O$_2$CH)], 90.74 [s, ($\underline{C}_5$H$_5$)Mo(PMe$_3$)$_2$(CO)(κ^1-O$_2$CH)], 174.20

[s, $CpMo(PMe_3)_2(CO)(\kappa^1\text{-}O_2\underline{C}H)$], 261.01 [t, $CpMo(PMe_3)_2(\underline{C}O)(\kappa^1\text{-}O_2CH)$, $^2J_{P\text{-}C} = 30$]. $^{31}P\{^1H\}$ NMR (C_6D_6): 20.96 [s, $CpMo(\underline{P}Me_3)_2(CO)(\kappa^1\text{-}O_2CH)$]. The ^{13}C labeled derivative $CpMo(PMe_3)_2(CO)(\kappa^1\text{-}O_2{}^{13}CH)$ was obtained in a similar manner employing $H^{13}CO_2H$.

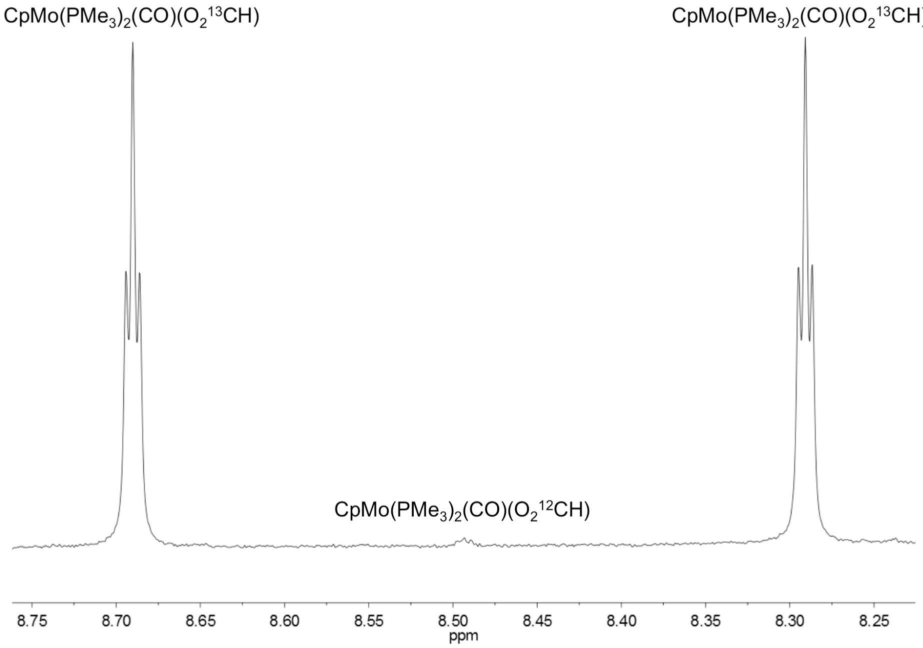

Figure 6. The ^1H NMR signal for ^{13}C enriched formate ligand of $CpMo(PMe_3)_2(CO)(\kappa^1\text{-}O_2{}^{13}CH)$, which exhibits coupling to both phosphorus ($^4J_{P\text{-}H} = 2$ Hz) and carbon ($^1J_{C\text{–}H} = 200$ Hz).

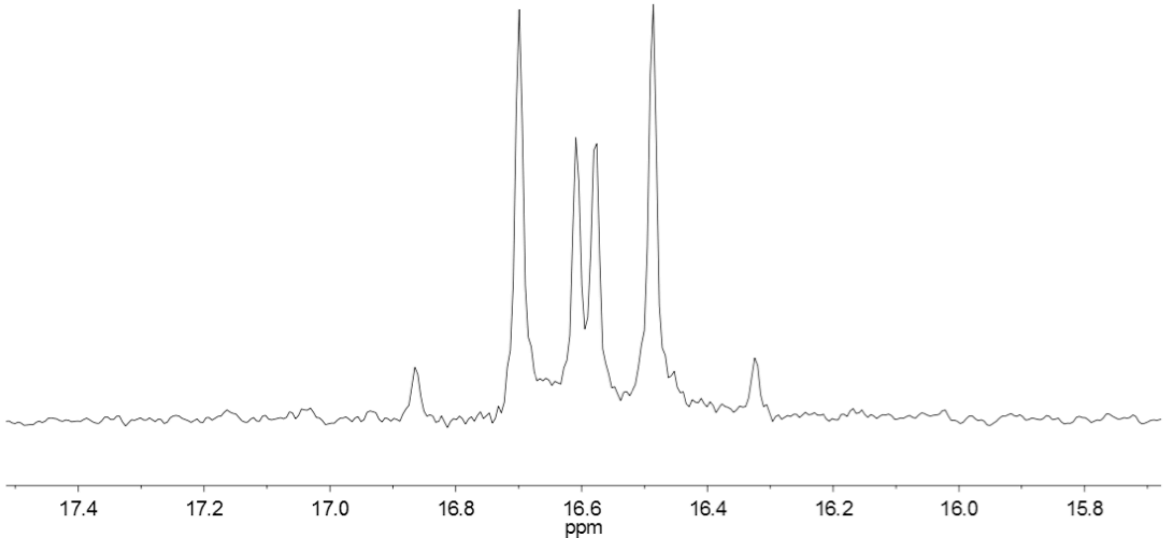

Figure 7. The $^{13}C\{^1H\}$ NMR signal for the PMe_3 ligands of $CpMo(PMe_3)_2(CO)(\kappa^1\text{-}O_2CH)$. The signal corresponds to an ABX spin system, and the observed spectrum can be simulated by $\Delta\delta_{PP} = 0.022$ ppm, $^2J_{P\text{-}P} = |25|$ Hz, $^1J_{P\text{-}C} = |27|$ Hz, and $^3J_{P\text{-}C} = 0$ Hz.[13]

2. Reactivity of $CpMo(PMe_3)_2(CO)H$ towards Formic Acid in Benzene

$CpMo(PMe_3)_2(CO)H$ (3.4 mg, 0.0099 mmol) was added to a solution of formic acid in C_6D_6 (0.64 mL of 0.39 M, 0.25 mmol) containing mesitylene (0.034 M, 0.022 mmol) as an internal standard in an NMR tube fitted with a J. Young valve. The resulting mixture was allowed to stand at room temperature and monitored *via* ^1H NMR spectroscopy over a period of days. While $CpMo(PMe_3)_2(CO)(\kappa^1\text{-}O_2CH)$ was observed initially by ^1H NMR spectroscopy at room temperature (*vide supra*), $CpMo(PMe_3)_2(CO)H$ became the primary species as catalysis proceeded and the formic acid was consumed.

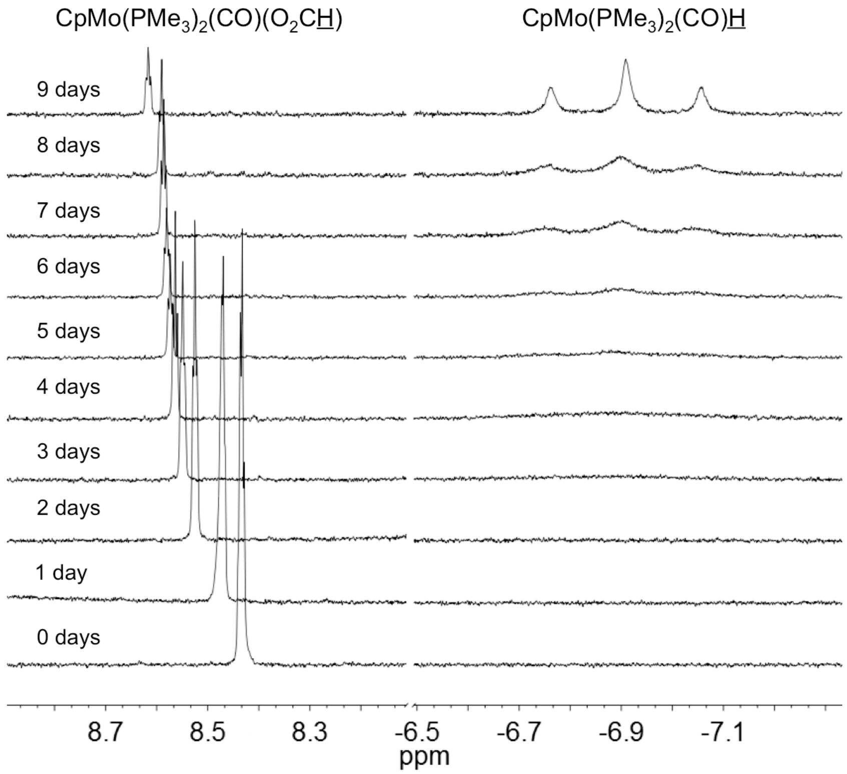

Figure 8. Change in the ^1H NMR signals of $CpMo(PMe_3)_2(CO)(\kappa^1\text{-}O_2C\underline{H})$ (left) and $CpMo(PMe_3)_2(CO)\underline{H}$ (right) as formic acid is consumed at room temperature. Initially, in the presence of a high concentration of formic acid, the molybdenum is largely in the form of the formate complex, $CpMo(PMe_3)_2(CO)(\kappa^1\text{-}O_2CH)$, whereas the hydride form, $CpMo(PMe_3)_2(CO)\underline{H}$, predominates at the end of the reaction when formic acid concentration is low. Note that the chemical shift of the formate moiety is influenced by the concentration of formic acid.

3. *Reactivity of $CpMo(PMe_3)_2(CO)H$ towards Formic Acid in Toluene at Low Temperature*

The reaction between $CpMo(PMe_3)_2(CO)H$ and formic acid was also examined at low temperature in d_8-toluene in order to provide evidence for a protonated species, $[CpMo(PMe_3)_2(CO)H_2]^+$. A solution of $CpMo(PMe_3)_2(CO)H$ (5.0 mg, 0.015 mmol) in d_8-toluene (*ca*. 0.7 mL) containing an excess of formic acid (0.15 mmol) in an NMR tube fitted with a J. Young valve was placed in the pre-cooled (198 K) probe of an NMR spectrometer. The sample was monitored by ^{1}H NMR spectroscopy while slowly increasing the temperature to 300 K. The formation of $[CpMo(PMe_3)_2(CO)H_2]^+$ was demonstrated by the observation of a triplet in the hydride region over the temperature range 225 – 276 K. ^{1}H NMR (d_8-toluene, 276K): –3.33 [t, 2H, $[CpMo(PMe_3)_2(CO)H_2]^+$, $^2J_{P\text{-}H} = 43$], 1.76 [br, 18H, $[CpMo(P(C\underline{H}_3)_3)_2(CO)H_2]^+$], 5.54 [s, 5H, $[(C_5\underline{H}_5)Mo(PMe_3)_2(CO)H_2]^+$]. $^{31}P\{^1H\}$ NMR (d_8-toluene, 276K): 8.86 [s, $CpMo(\underline{P}Me_3)_2(CO)H_2]^+$]. Upon warming to room temperature, the compound loses H_2 and transforms to $CpMo(PMe_3)_2(CO)(\kappa^1\text{-}O_2CH)$ (*vide supra*).

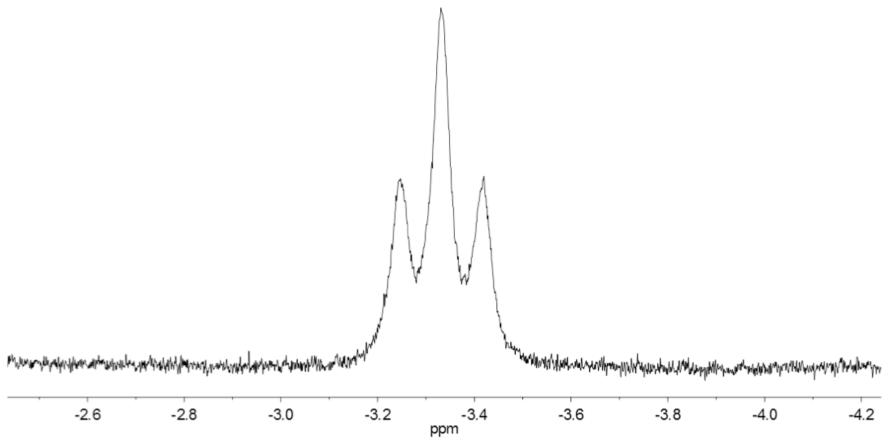

Figure 9. ^{1}H NMR spectrum (276 K) showing the hydride signal of $[CpMo(PMe_3)_2(CO)H_2]^+$ upon treatment of $CpMo(PMe_3)_2(CO)H$ with formic acid in d_8-toluene.

Comparison of Catalytic Activities for Dehydrogenation and Disproportionation of Formic Acid

The ability of $Cp^RMo(PMe_3)_{3-x}(CO)_xH$ (for $Cp^R = Cp$, $x = 0, 1, 2$ or 3; for $Cp^R = Cp^*$, $x = 1$, 2 or 3) to serve as catalysts for hydrogenation and disproportionation of formic acid was examined by NMR spectroscopy. For example, addition of $CpMo(CO)_3H$ (3.0 mg, 0.012 mmol) to a solution of $H^{13}CO_2H$ in C_6D_6 (0.64 mL of 0.39 M, 0.25 mmol) containing mesitylene (0.034 M, 0.022 mmol) results in the formation of carbon dioxide, methanol and methyl formate upon heating at 100˚C, as illustrated below.

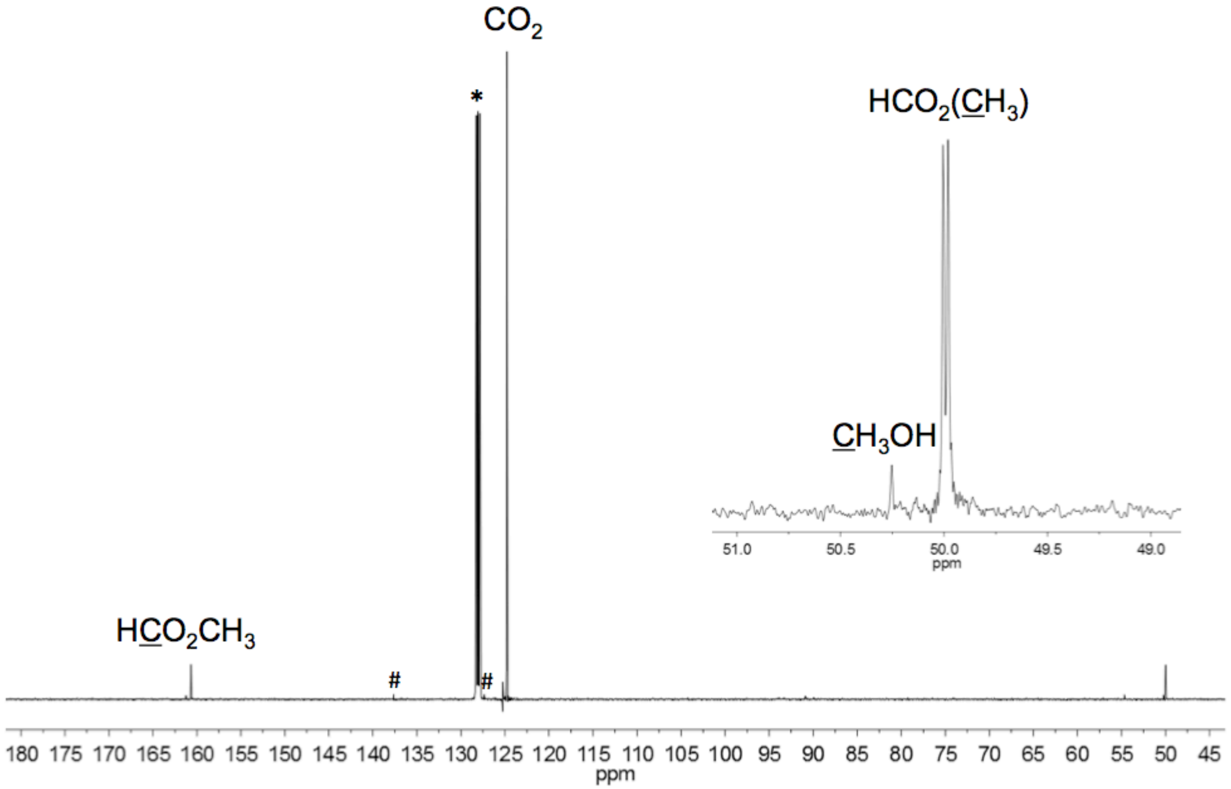

Figure 10. Catalytic formation of $H^{13}CO_2{}^{13}CH_3$ and $^{13}CH_3OH$ from $H^{13}CO_2H$ in C_6D_6 (*) by $CpMo(CO)_3H$, as demonstrated by ^{13}C NMR spectroscopy (# corresponds to a mesitylene standard). Spectrum recorded after four days at 100˚C.

The catalytic activities of $Cp^RMo(PMe_3)_{3-x}(CO)_xH$ were compared by addition of the catalysts (0.0099 mmol) to solutions of formic acid in C_6D_6 (0.64 mL of 0.39 M, 0.25

mmol) containing mesitylene (0.034 M, 0.022 mmol) as an internal standard in an NMR tube fitted with a J. Young valve (*Caution:* This reaction releases H_2 and CO_2, and appropriate safety precautions must be taken to avoid the buildup of high pressures). $CpMo(CO)_3H$, $Cp^*Mo(CO)_3H$, $CpMo(PMe_3)(CO)_2H$, $Cp^*Mo(PMe_3)(CO)_2H$ and $CpMo(PMe_3)_3H$ displayed no appreciable catalysis at room temperature, and so the reactivity was monitored at 100˚C (Table 1). $CpMo(PMe_3)_2(CO)H$ and $Cp^*Mo(PMe_3)_2(CO)H$ are competent catalysts at room temperature and 100 ˚C, and were monitored at both temperatures (Table 1).

The overall conversion of formic acid is a composite of dehydrogenation (to form CO_2 and H_2) and disproportionation (to form MeOH and HCO_2Me), and this was taken into account when evaluating the individual catalytic efficiencies. Specifically, the amount of formic acid that undergoes dehydrogenation is given by the expression $[HCO_2H]_{dehydrogenated} = [HCO_2H]_{consumed} - \{3 \times [MeOH]_{formed}\} - \{4 \times [HCO_2Me]_{formed}\}$, and the turnover number (TON) for dehydrogenation is defined as $[HCO_2H]_{dehydrogenated}/[Cp^RMo(PMe_3)_{3-x}(CO)_xH]_{initial}$. Turnover frequencies (TOF) are reported for 50% conversion and are calculated by dividing the TON at this point by the time required to achieve this value (Table 1). Selectivity for formation of methanol/methyl formate is defined as the percentage of formic acid used to produce methanol/methyl formate and is equal to $3 \times \{[MeOH]_{formed} + [HCO_2Me]_{formed}\}/\{[HCO_2H]_{consumed} - [HCO_2Me]_{formed}\} \times 100$ (Table 2). The selectivity as a function of formic acid and catalyst concentration was measured as summarized in Table 3. The indicated amounts of formic acid and $CpMo(CO)_3H$ were added to C_6D_6 (0.64 mL) with mesitylene (3.0 μL, 0.022 mmol) as an internal standard in an NMR tube fitted with a J. Young valve. Reactions were heated at 100 ˚C and were monitored periodically via ^1H NMR spectroscopy.

Table 1. Turnover frequencies (h^{-1}) for dehydrogenation of formic acid by $Cp^RMo(PMe_3)_{3-x}(CO)_xH$ at 100°C.[a]

	**Cp**	**Cp***
$Cp^RMo(PMe_3)_2(CO)H$	31 (0.77)[b]	54 (1.8)[b]
$Cp^RMo(PMe_3)(CO)_2H$	0.64	1.2
$Cp^RMo(CO)_3H$	0.67	0.33

(a) $[Cp^RMo(PMe_3)_{3-x}(CO)_xH]$ = 0.016 M, $[HCO_2H]_{initial}$ = 0.39 M, C_6D_6, values at 50% conversion.

(b) Values in parentheses are for catalysis performed at room temperature.

Table 2. Selectivities for disproportionation *versus* dehydrogenation of formic acid by $Cp^RMo(PMe_3)_{3-x}(CO)_xH$ at 100°C.[a]

	**Cp**	**Cp***
$Cp^RMo(PMe_3)_2(CO)H$	1.2	1.2
$Cp^RMo(PMe_3)(CO)_2H$	4.0	2.1
$Cp^RMo(CO)_3H$	11.1	10.6

(a) $[Cp^RMo(PMe_3)_{3-x}(CO)_xH]$ = 0.016 M, $[HCO_2H]_{initial}$ = 0.39 M, C_6D_6, values at 100% conversion.

Table 3. Selectivities for disproportionation *versus* dehydrogenation of formic acid by $CpMo(CO)_3H$ as a function of catalyst and formic acid concentration.[a]

**$[CpMo(CO)_3H]$ (M)**	**$[HCO_2H]$ (M)**	**Selectivity (%)**
0.015	0.049	6.9
0.014	0.18	9.6
0.014	0.36	15
0.014	0.67	10
0.041	0.36	12
0.10	0.36	15
0.025	0.34	21

(a) 100˚C, C_6D_6, values at 100% conversion.

Reactivity of $CpMo(CO)_3H$ towards Formic Acid in the Presence of D_2

$CpMo(CO)_3H$ (5.0 mg, 0.020 mmol) and $H^{13}CO_2H$ (8.0 μL, 0.21 mmol) were added to C_6D_6 or C_6H_6 (*ca.* 0.7 mL) in two NMR tubes equipped with J. Young valves. The tubes were degassed *via* a freeze-pump-thaw cycle, and exposed to D_2 (1 atm). The reactions were heated at 100˚C (side-by-side) and monitored by NMR spectroscopy (^{1}H and ^{13}C for the C_6D_6 solution and ^{2}H NMR for the C_6H_6 solution), which demonstrated that there was no significant deuterium incorporation into either the methanol or methyl formate.

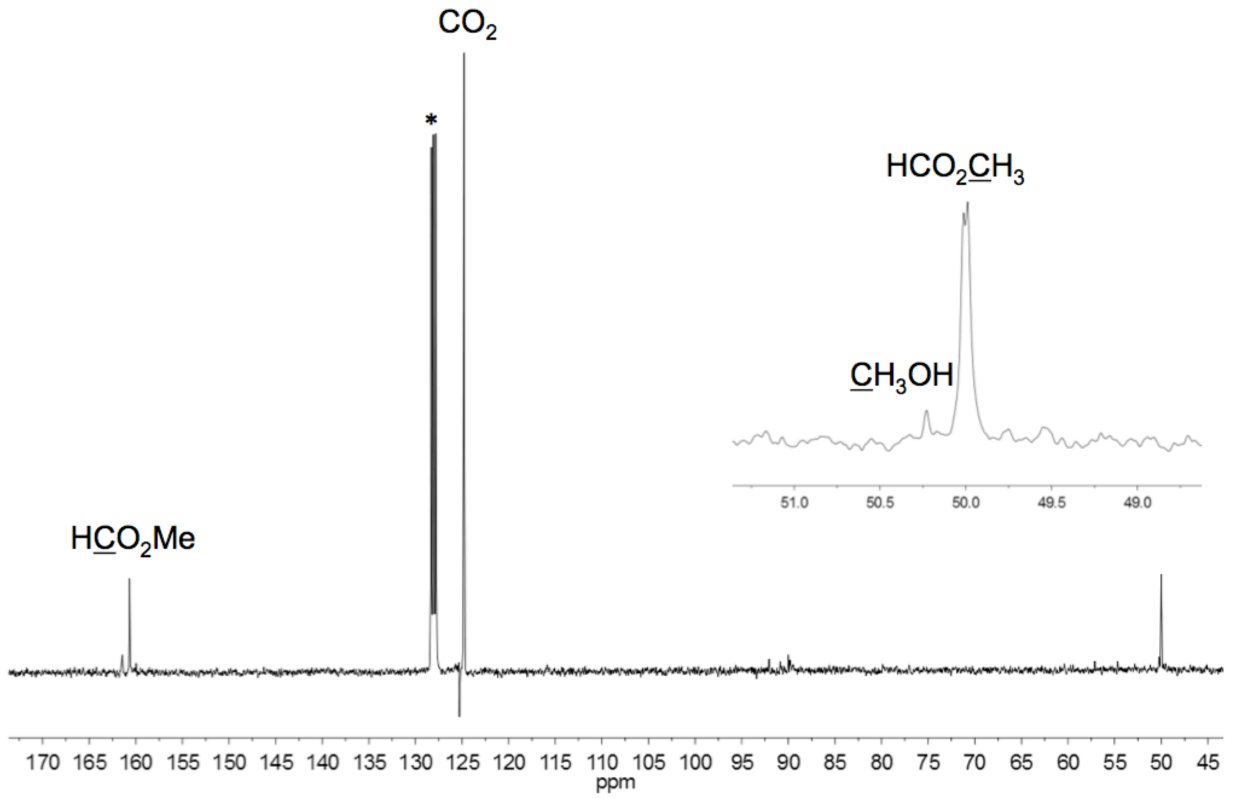

Figure 11. Catalytic formation of HCO_2Me and MeOH from $H^{13}CO_2H$ in the presence of D_2 as demonstrated by ^{13}C NMR spectroscopy (* = C_6D_6).

General Procedure for the Transfer Hydrogenation of Carbonyls with Formic Acid

A solution of formic acid in C_6D_6 (0.64 mL of 0.39 M, 0.25 mmol) containing mesitylene as an internal standard (0.034 M, 0.022 mmol) was treated sequentially with RC(O)R′ and $CpMo(CO)_3H$ (2.4 mg, 0.0098 mmol), where RC(O)R′ = MeCHO, Pr^iCHO, RC(O)Me (R = Me, Pr^i, Bu^t, Ph) and Ph_2CO. The reactions were heated at 100 °C and monitored *via* ^1H NMR spectroscopy until the formic acid was completely consumed. Selectivity for transfer hydrogenation is defined as the percentage of formic acid used to hydrogenate the C=O bonds: $\{[ROH]_{formed} + [HCO_2R]_{formed}\} / \{[HCO_2H]_{consumed} - [HCO_2R]_{formed} - [HCO_2Me]_{formed}\} \times 100$ and the values are listed in Table 4.

Table 4. Selectivity for transfer hydrogenation of carbonyl compounds with formic acid by $CpMo(CO)_3H$ at 100°C[a]

Substrate	Selectivity (%)
MeCHO[b]	34
Pr^iCHO	32[c]
MeCOMe	19
Pr^iCOMe	18
Bu^tCOMe	19
PhCOMe	22
PhCOPh	29[c]

(a) $[CpMo(CO)_3H] = 0.016$ M, $[HCO_2H]_{initial} = [RCOR']_{initial} = 0.39$ M, C_6D_6, values at 100% conversion.

(b) $[CpMo(CO)_3H] = 0.016$ M, $[HCO_2H]_{initial} = 0.39$ M, $[MeCHO]_{initial} = 1.3$ M, C_6D_6, value at 100% conversion.

(c) Selectivities are the average of two runs.

Reaction of $CpMo(PMe_3)_2(CO)H$ with CO_2 at High Pressure

A solution containing $CpMo(PMe_3)_2(CO)H$ (0.023 M) and mesitylene (0.034 M) as an internal standard in C_6D_6 (*ca.* 0.64 mL) was placed in a sapphire NMR tube, which was subsequently charged with CO_2 (59.9 atm). The solution was monitored by ^1H NMR spectroscopy, thereby indicating the generation of $CpMo(PMe_3)_2(CO)(\kappa^1\text{-}O_2CH)$ as a component of an equilibrium mixture after a period of two days. The equilibrium constant was determined using the relative ratio of $CpMo(PMe_3)_2(CO)H$ and $CpMo(PMe_3)_2(CO)(\kappa^1\text{-}O_2CH)$, and the pressure of CO_2, such that $K = [CpMo(PMe_3)_2(CO)H]/([CpMo(PMe_3)_2(CO)(\kappa^1\text{-}O_2CH)] \times P_{CO_2}) = 0.01\ atm^{-1}$.

Reactivity of $CpMo(CO)_3H$ Towards Formic Acid

$CpMo(CO)_3H$ (5.0 mg, 0.020 mmol) was added to a solution of formic acid (7.7 μL, 0.20 mmol) in d_8-THF (*ca.* 0.7 mL) in an NMR tube equipped with a J. Young valve and monitored by ^1H NMR spectroscopy, thereby indicating that there was no reaction at either room temperature or upon heating at 100˚C for four days.

Table 5. Crystal, intensity collection, and refinement data.

	**$[CpMo(PMe_3)_3H_2][Cl]$**
lattice	Orthorhombic
formula	$C_{14}H_{34}ClMoP_3$
formula weight	426.71
space group	$P2_12_12_1$
a/ Å	11.8282(14)
b/ Å	12.4490(15)
c/ Å	13.6005(16)
α/ °	90
β/ °	90
γ/ °	90
V/ Å^3	2002.7(4)
Z	4
temperature (K)	130(2)
radiation (λ, Å)	0.71073
ρ (calcd.) g cm^{-3}	1.415
μ (Mo Kα), mm^{-1}	1.017
θ max, deg.	30.73
no. of data collected	27188
no. of data	6204
no. of parameters	189
R_1 [$I > 2\sigma(I)$]	0.0550
wR_2 [$I > 2\sigma(I)$]	0.0949
R_1 [all data]	0.0926
wR_2 [all data]	0.1069
GOF	1.005
R_{int}	0.1010

REFERENCES

(1) (a) McNally, J. P.; Leong, V. S.; Cooper, N. J. in *Experimental Organometallic Chemistry*, Wayda, A. L.; Darensbourg, M. Y., Eds.; American Chemical Society: Washington, DC, 1987; Chapter 2, pp 6-23.

(b) Burger, B.J.; Bercaw, J. E. in *Experimental Organometallic Chemistry*; Wayda, A. L.; Darensbourg, M. Y., Eds.; American Chemical Society: Washington, DC, 1987; Chapter 4, pp 79-98.

(c) Shriver, D. F.; Drezdzon, M. A.; *The Manipulation of Air-Sensitive Compounds*, 2nd Edition; Wiley-Interscience: New York, 1986.

(2) Fulmer, G. R.; Miller, A. J. M.; Sherden, N. H.; Gottlieb, H. E.; Nudelman, A.; Stoltz, B. M.; Bercaw, J. E.; Goldberg, K. I. *Organometallics* **2010**, *29*, 2176-2179.

(3) *Nuclear Magnetic Resonance Spectroscopy,* Nelson, J. H.; Prentice Hall: New Jersey, 2003; p 79.

(4) (a) Fischer, E. O. *Inorg. Synth.* **1963**, *7*, 136-139.

(b) King, R. B. in *Organometallic Syntheses, Volume 1: Transition Metal Compounds*, Eisch, J. J.; King, R. B., Eds.; Academic Press, Inc.: New York, 1965; Chapter H.1, pp 156-158.

(c) Bruchell, R. P. L.; Sirsch, P.; Decken, A.; McGrady, G. S. *Dalton Trans.* **2009**, *30*, 5851-5857.

(5) (a) Kalck, P.; Pince, R.; Poilblanc, R. *J. Organomet. Chem.* **1970**, *24*, 445-452.

(b) Alt, H. G.; Engelhardt, H. E.; Kläui, W.; Müller, A. *J. Organomet. Chem.* **1987**, *331*, 317-327.

(6) (a) Brookhart, M.; Cox, K.; Cloke, F. G. N.; Green, J. C.; Green, M. L. H.; Hare, P. M. *J. Chem. Soc. Dalton Trans.* **1985**, 423-433.

(b) Abugideiri, F.; Kelland, M. A.; Poli, R.; Rheingold, A. L. *Organometallics* **1992**, *11*, 1303-1311.

(7) Kubas, G. J.; Kiss, G.; Hoff, C. D. *Organometallics* **1991**, *10*, 2870–2876.

(8) Tudoret, M.-J.; Robo, M.-L.; Lapinte, C. *Organometallics* **1992**, *11*, 1419–1422.

(9) Shin, J. H.; Churchill, D. G.; Parkin, G. *J. Organomet. Chem.* **2002**, *642*, 9-15.

(10) (a) Sheldrick, G. M. SHELXTL, An Integrated System for Solving, Refining, and Displaying Crystal Structures from Diffraction Data; University of Göttingen, Göttingen, Federal Republic of Germany, 1981.

(b) Sheldrick, G. M. *Acta Cryst.* **2008**, *A64*, 112-122.

(11) (a) Le Gall, J.-Y.; Kubicki, M. M.; Petillon, F. Y. *J. Organomet. Chem.* **1981**, *221*, 287–290.

(b) Marsella, J. A.; Huffman, J. C.; Caulton, K. G.; Longato, B.; Norton, J. R. *J. Am. Chem. Soc.* **1982**, *104*, 6360–6368.

(12) The low temperature ^{1}H NMR spectrum of $[CpMo(PMe_3)_3H_2][O_2CH]$ is very similar to that of $[CpMo(PMe_3)_3H_2][BF_4]$. See: Fettinger, J. C.; Kraatz, H.-B.; Poli, R.; Quadrelli, E. A.; Torralba, R. C. *Organometallics* **1998**, *17*, 5767-5775.

(13) For a closely related example, see: Sattler, A.; Parkin, G. *J. Am. Chem. Soc.* **2012**, *124*, 2355-2366.
